# The von Willebrand factor stamps plasmatic extracellular vesicles from glioblastoma patients

**DOI:** 10.1038/s41598-021-02254-7

**Published:** 2021-11-23

**Authors:** Quentin Sabbagh, Gwennan André-Grégoire, Carolina Alves-Nicolau, Aurélien Dupont, Nicolas Bidère, Emmanuel Jouglar, Laëtitia Guével, Jean-Sébastien Frénel, Julie Gavard

**Affiliations:** 1 Team SOAP, Signaling in Oncogenesis Angiogenesis and Permeability, CRCINA, Inserm, CNRS, Universite de Nantes, IRS-UN blg, Room 402, 8 quai Moncousu, 44000 Nantes, France; 2 Integrated Center for Oncology, Institut de Cancerologie de l’Ouest, St. Herblain, France; 3 Univ Rennes, CNRS, Inserm, BIOSIT, UMS 3480, US_S 018, 35000 Rennes, France

**Keywords:** Cancer, Cell biology

## Abstract

Glioblastoma is a devastating tumor of the central nervous system characterized by a poor survival and an extremely dark prognosis, making its diagnosis, treatment and monitoring highly challenging. Numerous studies have highlighted extracellular vesicles (EVs) as key players of tumor growth, invasiveness and resistance, as they carry and disseminate oncogenic material in the local tumor microenvironment and at distance. However, whether their quality and quantity reflect individual health status and changes in homeostasis is still not fully elucidated. Here, we separated EVs from plasma collected at different time points alongside with the clinical management of GBM patients. Our findings confirm that plasmatic EVs could be separated and characterized with standardized protocols, thereby ensuring the reliability of measuring vesiclemia, *i.e.* extracellular vesicle concentration in plasma. This unveils that vesiclemia is a dynamic parameter, which could be reflecting tumor burden and/or response to treatments. Further label-free liquid chromatography tandem mass spectrometry unmasks the von Willebrand Factor (VWF) as a selective protein hallmark for GBM-patient EVs. Our data thus support the notion that EVs from GBM patients showed differential protein cargos that can be further surveyed in circulating EVs, together with vesiclemia.

## Introduction

Glioblastoma (GBM) is the most common primary malignant brain tumors in adults, and the most aggressive one, making it a major therapeutic challenge. Occurring in 70% of the cases between 45 and 70 years-old patients, prognosis remains extremely poor despite standardized, combative treatment. Indeed, tumor relapse is almost inevitable 7 to 10 months post-therapy, while the median survival is estimated at 14 months and the 5-year survival rate is about 5%. The actual frontline standard reference treatment has been established by Stupp et al*.* in 2005 and combines debulking surgery (if possible), followed by a 6-week adjuvant radiochemotherapy followed with a 6-month chemotherapy, both based on standardized doses of temozolomide (TMZ), an alkylating agent^1^. Despite this harsh therapeutic regimen, GBM recurrence is rapid and fatal. In this context of therapeutic impasse, new strategies are needed.

Numerous studies have unveiled the central role of Extracellular Vesicles (EVs) as key mediators of intercellular communication in the GBM microenvironment^2–8^. The term EVs is a moniker for a variety of small, heterogeneous, membrane vesicles (30–1000 nm), released by virtually all cells into the surrounding milieu and consecutively circulating in biofluids, such as blood, urine etc.…^9^. These lipid bilayer particles transport protected bioactive materials i.e. proteins, lipids, and nucleic acids from their cell of origin to recipient cells, located either in the vicinity or at distance^10^. EVs therefore act as potent mediators for cell communication in tissue and throughout fluids and body. Indeed, the EV secretory pathway is suspected to be perverted by both tumor and stromal cells, and might distribute oncogenic material and non-physiological information^2,5,6,8,10,11^. Thereby, EVs have been suggested to serve as major communication tools from and towards stem-like tumor cells, differentiated tumor cells, immune system and vascular endothelial cells, to support tumor growth, invasiveness and survival^2^. Beside their instrumental role in transcellular message delivery, EVs quantitatively and qualitatively may vary with gender, ageing, and during illness^12,13^. EVs may thus represent a valuable, informative resource on health status and disease progression. Vesiclemia, *i.e.* EV quantity in a given volume, can be obtained from minimally invasive liquid biopsies and is currently scrutinized in a wide variety of diseases^12^. However, whether vesiclemia reflects patho-physiological changes is still not fully elucidated. Here, we established reproducible, standardized methodologies to isolate EVs from plasma, collected at different time points during the clinical management of GBM patients, and stored from two biobanks. Our longitudinal analysis unveils that vesiclemia is a dynamic parameter, which could inform on tumor burden and/or response to treatments. Further proteomic analysis unmasks the von Willebrand Factor (VWF) as a selective protein hallmark for GBM-patient separated EVs.

## Results

### Separation of extracellular vesicles from peripheral blood

**Figure 1:**
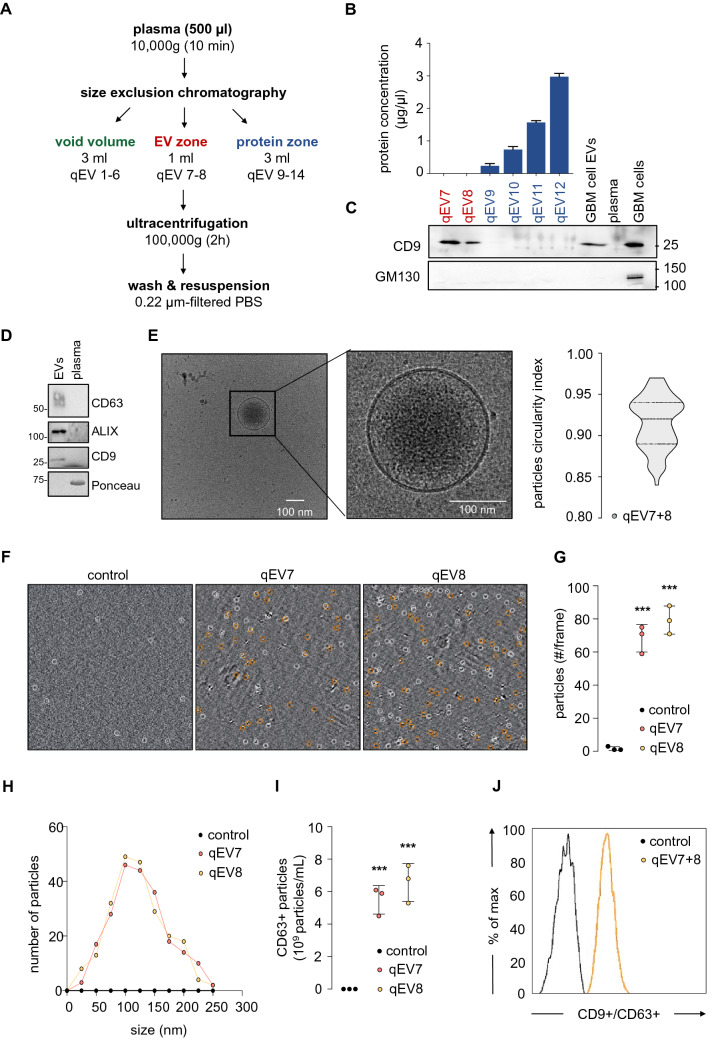
Separation of extracellular vesicles from human peripheral blood. (**A**) Diagram representing the separation of extracellular vesicles (EVs) from peripheral blood. (**B**) Total protein concentrations were measured in each size-exclusion chromatography (SEC) lysed fractions from qEV7 to qEV12. (**C**) Equal volume of protein lysates of qEV fractions were separated by SDS-PAGE and analyzed by immunoblot for CD9 (specific EV marker) and GM130 (putative intracellular membrane protein contaminant). EVs from GSC-conditioned media and total cell lysate (TCL) serve as controls. (**D**) Protein lysates of qEV7-8 pooled fractions from one healthy donor (EVs) and the corresponding plasma were separated by SDS-PAGE and analyzed by immunoblot for specific EV markers (CD9 and CD63 tetraspanins and ALIX). Blots are representative of n > 3. (**E**) Cryo-transmission electron microscopy (cryo-TEM) was deployed to image EVs in pooled qEV7-8 fractions and to estimate sample purity (left panel). Nanoparticle morphology was evaluated using circularity index in n > 70 cryo-TEM images (right panel). (**F**, **G**) Quantitative analysis of EVs was performed using interferometry light microscope (ILM), which tracks particles based on Brownian motion, in qEV7 and qEV8 fractions. (**H**) Similarly, diameter distribution was monitored in representative qEV7 and qEV8 fractions. (**I**) EV abundance was estimated via CD63 ELISA. (**J**) Expression of CD63 was estimated by flow cytometry, following CD9-positive immunocapture in the qEV7 and qEV8 fractions. Filtered PBS was used as control (**F**–**J**). Data are representative of at least 3 independent experiments. ANOVA, ***p < 0.001. S.E.M. are shown in panels G and I.

Standardized, validated, and complementary procedures were implemented^14,15^ in order to isolate extracellular vesicles (EVs) from plasma. As shown in Fig. 1A, EV-containing fractions were collected through a low recovery/high specificity procedure using size exclusion chromatography (SEC), from a volume of 500 µl of 10,000*g* centrifugated plasma, using an automatic fraction collector. Size-based separated EV fractions were recovered by ultracentrifugation at 100,000*g* for 2 h, resuspended in 0.22 µm-filtered PBS. To first evaluate the EV preparation degree of purity, the nature, and the extent of the protein cargo were estimated (Fig. 1B). While an ascending concentration of proteins, most likely from bloodstream origin, was measured in the discarded fractions (qEV9-12), protein concentration was below the detectable levels in qEV7 and qEV8 fractions, disqualifying a massive extravesicular protein contamination in the eluted EVs (Fig. 1B, qEV7-8). Next, western-blot analysis shows the prominent presence in the EV fractions of the EV-associated transmembrane tetraspanin CD9, which was lost in later fractions (Fig. 1C). Conversely, the Golgi protein marker GM130 was absent, excluding potential co-isolation of cellular components and debris (Fig. 1C). Accordingly, pooled qEV7 and qEV8 fractions contain typical markers of EVs, namely Alix, CD9, and CD63, while exempted from strong plasmatic protein contamination (Fig. 1D). We further documented the quality of separated EVs with cryo-transmission electron microscopy (cryo-TEM, Fig. 1E). Large fields did not unmask massive aggregate contamination. Instead, typical lipid bilayer vesicles with a mean diameter of around 130 nm were detected. These electron-dense materials identified small EVs with circularity index of around 0.91, suggesting their integrity (Fig. 1E). Likewise, plasmatic EVs were visualized with single particle tracking using interferometry light microscopy (ILM) technology (Videodrop, Myriade, Paris, France), which enables the estimation of the concentration of particles according to Brownian motion (Fig. 1F). The amount of EVs was readily detected in the qEV7 and qEV8 fractions with approximately 70 and 80 particles tracked per frame (orange circles), respectively, and a mean size of around 100–110 nm, as compared to filtered PBS negative control (Fig. 1F–H). Likewise, the presence of EVs in both qEV7 and qEV8 fractions was underlined through ELISA detection of the tetraspanin transmembrane EV passenger CD63 (Fig. 1I). We found an elevated level of CD63-positive particles, 5.5 × 10^9^ and 6.3 × 10^9^ particles/ml, in qEV7 and qEV8 fractions, respectively, as compared to 0.22 µm-filtered PBS used as a negative control (Fig. 1I). These results were corroborated using flow cytometry detection of CD63-positive particles separated with anti-CD9 capture-beads (Fig. 1J). Together, these findings confirm that plasmatic EVs could be separated and characterized with standard protocols, thereby ensuring the reliability of measuring vesiclemia.

### Vesiclemia evolves along glioblastoma progression

**Figure 2:**
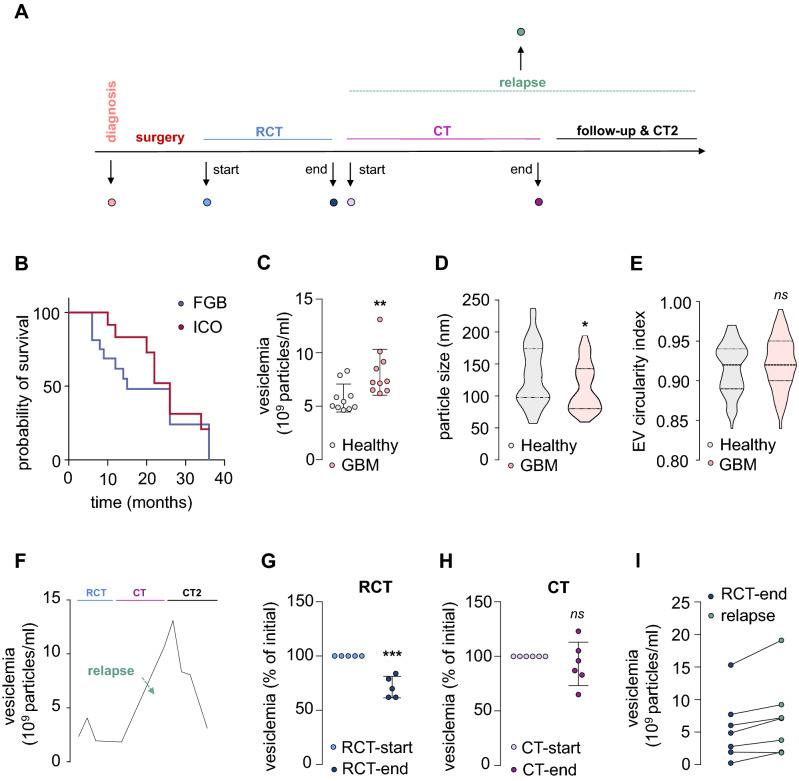
Vesiclemia evolves along glioblastoma progression. (**A**) Diagram of glioblastoma (GBM) management according to Stupp et al. protocol^1^. RCT: radiochemotherapy, CT: chemotherapy. (**B**) Kaplan–Meier survival curves of both exploited biocollections from the French Glioblastoma Biobank (FGB) and Integrated Center for Oncology (ICO), with 20 and 10 patients, respectively. (**C**) Vesiclemia (number of particles/ml) was measured by interferometry light microscope (ILM) in healthy donors and GBM patients (n = 10 in each group). (**D**, **E**) Analysis of plasmatic EV mean size in healthy donors and GBM patients using cryo-electron microscopy (cryo-TEM), n > 65 for each group. Alternatively, particle morphology (circularity index) was evaluated using in cryo-TEM images. (**F**–**I**) Vesiclemia was measured via CD63 ELISA. Evolution of the vesiclemia along the follow-up of one patient throughout tumor management from Stupp protocol (RCT and CT) to second line CT2 (Bevacizumab) (**F**). Vesiclemia was measured in longitudinal samples from several GBM patients, in order to assess the impact of radio-chemotherapy (RCT) (n = 5) (**G**), chemotherapy (n = 6) (**H**), and relapse (n = 7) (**I**). Mann–Whitney test, *p < 0.05, **p < 0.01, ***p < 0.001. S.E.M. are shown in panels C, G, and H.

To tackle the question whether quantity and/or quality of circulating EVs could represent potential hallmark of GBM evolution, we performed a longitudinal analysis of the vesiclemia (*i.e.* concentration of particles/ml) throughout tumor management. In this purpose, two biocollections of plasmatic samples from GBM patients were harnessed, namely the French Glioblastoma Biobank (FGB), composed of multicentric samples collected upon diagnosis, and, the Integrated Center for Oncology longitudinal biocollection (ICO) with peripheral blood sequentially collected alongside their care (*i.e.* throughout the Stupp et al*.* protocol^1^, during the follow-up, and at tumor relapse) (Fig. 2A). Table 1 reported the clinical information on the 30 patients enrolled from the two biobanks, including 20 at diagnosis and 10 in longitudinal cohort, with 15- and 26-month median survival, respectively (Fig. 2B). Plasmatic EVs were separated by size exclusion chromatography (SEC) and the vesiclemia was estimated either by single particle tracking using either ILM technology or ELISA for CD63.

**Table 1:** Clinical information from GBM patients regarding age at diagnosis, gender, time to death and therapeutical care.

Biobank	Age	Gender	Elapsed time to death	Surgery	RCT	2nd line
FGB#1	61	Female	12	Partial	Yes	n/a
FGB#2	71	Male	6	Total	Yes	n/a
FGB#3	64	Female	8	Biopsy	Yes	n/a
FGB#4	69	Male	9	Total	Yes	Bevacizumab
FGB#5	74	Male	13	Total	Yes	Bevacizumab
FGB#6	69	Male	6	Biopsy	Yes	n/a
FGB#7	60	Female	15	Total	Yes	Lomustine
FGB#8	72	Male	6	Total	Yes	Irinotecan
FGB#9	64	Male	38	Total	Yes	Irinotecan
FGB#10	69	Female	26	Total	Yes	Bevacizumab
FGB#11	46	Male	12	Total	Yes	Bevacizumab
FGB#12	55	Male	8	Total	Yes	n/a
FGB#13	81	Male	13	Total	Yes	n/a
FGB#14	63	Female	15	Biopsy	Yes	Lomustine
FGB#15	66	Male	7	Biopsy	Yes	n/a
FGB#16	65	Male	15	Total	Yes	n/a
FGB#17	51	Male	17	Total	Yes	Bevacizumab
FGB#18	74	Female	3	Partial	yes	n/a
FGB#19	48	Male	3	Total	Yes	Bevacizumab
FGB#20	68	Male	14	Partial	Yes	n/a
ICO#1	52	Female	26	Total	Yes	Temozolomide
ICO#2	51	Female	24	Total	Yes	Lomustine
ICO#3	67	Male	18	Total	Yes	Bevacizumab
ICO#4	79	Male	30	Biopsy	Yes	Bevacizumab
ICO#5	67	Female	20	Partial	Yes	Bevacizumab
ICO#6	63	Male	Alive	Total	Yes	n/a
ICO#7	77	Female	4	Biopsy	Yes	Bevacizumab
ICO#8	55	Male	Alive	Partial	Yes	Bevacizumab
ICO#9	64	Male	20	Biopsy	Yes	Lomustine
ICO#10	79	Male	8	Partial	Yes	n/a

Two cohorts were used here (FGB and ICO, as described in the method section). Elapsed time from diagnosis to death is expressed in months.

We firstly compared the vesiclemia of 10 newly diagnosed GBM patients and 10 healthy donors using interferometry microscopy. A significantly higher vesiclemia was found in the tumor patient group with a mean concentration about 8.5 × 10^9^ particles/ml, as compared to healthy donors with 5.9 × 10^9^ particles/ml, corroborating earlier findings^4^ (Fig. 2C). Moreover, electron microscopy analysis demonstrated that plasmatic EVs from GBM patients upon diagnosis are significantly smallest with a mean size of 101 nm, in comparison to the non-tumor donor group, exhibiting a mean size of 129 nm (Fig. 2D), while sharing similar circular morphology (Fig. 2E). The reduction in the EV size might reflect changes in the release pathway and/or the tissular origin of circulating nanoparticles.

Vesiclemia was measured in ten GBM patients alongside their treatment. To illustrate this longitudinal analysis, data from one patient with conventional clinical features^1^, in terms of administered treatments and time to relapse and demise, are presented here (Table 1, Fig. 2F). This personalized analysis highlights that this selected patient’s vesiclemia varies upon tumor progression, with a noticeable peak at the relapse period that occurs in this clinical case during the course of the 6-month temozolomide chemotherapy (CT). To further investigate how treatments might impact this biological parameter, we then explored the effect of the initial radiochemotherapy (RCT) on the plasmatic EV abundance of 5 patients sampled prior to and during RCT. Remarkably, all 5 patients displayed a drop of the vesiclemia during the treatment with a significant decrease of about 35%, outlining the impact of the RCT on the quantity of circulating plasmatic EVs (Fig. 2G). Furthermore, we monitored the evolution of the vesiclemia during the 6-month temozolomide chemotherapy (CT), typically administered after 1-month break following RCT. The plasmatic EV abundance of 6 patients at the beginning (months 1–2) and the end (months 5–6) of the chemotherapy was not obviously modified over the treatment period (Fig. 2H). Finally, the chronological evolution of the plasmatic EV abundance was evaluated for 7 relapsing patients, *i.e.* comparing values obtained at the initial RCT and at the time the tumor relapsed (Table 1). Noticeably, 6 out of these 7 patients unveiled an increase of about 40% of their vesiclemia when the tumor recurs, outlining a potential correlation between the plasmatic EV concentration and GBM relapse (Fig. 2I). Therefore, these findings highlight the vesiclemia as a variable and dynamic biological parameter that might be regulated by several therapeutic factors, including radiations and drug administration, while placing the longitudinal analysis of the vesiclemia as a potential aid to anticipate patient follow-up and/or confirm tumor relapse. However, larger cohorts and longitudinal analyses of the evolution of the circulating EV cargo throughout tumor management are needed.

### Proteomic analysis of circulating EVs from GBM patients unveils specific cargos

**Figure 3:**
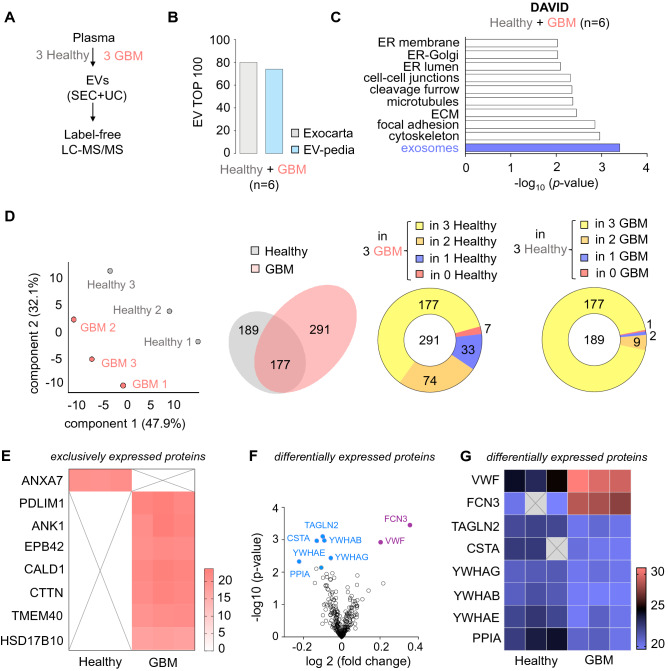
Proteomic analysis of circulating EVs from GBM patients unveils specific cargos. (**A**) Plasmatic extracellular vesicles (EVs) were separated from six plasmas (3 healthy donors and 3 GBM patients), as described in Fig. 1A. Protein cargoes were quantitatively analyzed through label-free liquid chromatography tandem mass spectrometry (LC–MS/MS). (**B**) Representation of the top 100 EV-enriched proteins estimated with Exocarta and EVpedia databases, when combining proteomic analysis from 6 samples. (**C**) DAVID analysis of the top Gene Ontology (GO) functions from the 6 analyzed samples. (**D**) Principal component analysis of healthy donors and GBM patient samples (left panel). Venn diagram of the detected proteins and their repartition between healthy donor and GBM patient groups (middle and right panel). (**E**) Heatmap analysis of exclusively expressed proteins. (**F**, **G**) Volcano plot and heatmap analysis of differentially expressed proteins.

In order to get qualitatively insights on circulating EVs, label-free liquid chromatography tandem mass spectrometry was performed on EV fractions from 6 randomized plasmas from GBM patients (n = 3) and healthy donors (n = 3) (Fig. 3A, Supplemental Table S1). Their cargo reached 80% and 74% of enriched EV proteins, when compared to established Exocarta and Vesiclepedia databases, respectively (Fig. 3B). Further exploration of the database for annotation, visualization and integrated discovery (DAVID) highlighted “exosome” as the top Gene Ontology (GO) functions in plasma EV proteome, together with other dynamic processes, such as “cytoskeleton”, “adhesion” and “endoplasmatic reticulum functions” (Fig. 3C). Principal component analysis (PCA) allowed to partition two distinct populations, grouping EV-protein cargo from GBM patients on one side and EV-protein cargo from healthy donors on the other (Fig. 3D). In total, 291 and 189 proteins were detected within the three samples of each group, GBM patients and donors, respectively. A large part of them were shared between groups (*i.e.* 177 proteins, Fig. 3D). Of note, a very few were exclusive to each group, namely 1 in the donor group (ANXA7) and 7 in the GBM group (PDLIM1, ANK1, EPB42, CALD1, CTTN, TMEM40, HSD17B10) (Fig. 3D, E, Supplemental Table S2). Likewise, differentially expressed proteins were visualized on Volcano’ plot (Fig. 3F, Supplemental Table S3). Heatmap unmasked 6 proteins significantly under-represented (TAGLN2, CSTA, YWHAG, YWHAB, YWHAE, and PPIA) in EVs from GBM patients, as compared to the ones from healthy donors (Fig. 3G). Conversely, 2 proteins, namely VWF and FCN3, were detected as more abundant in EVs from GBM patients (Fig. 3G).

**Figure 4:**
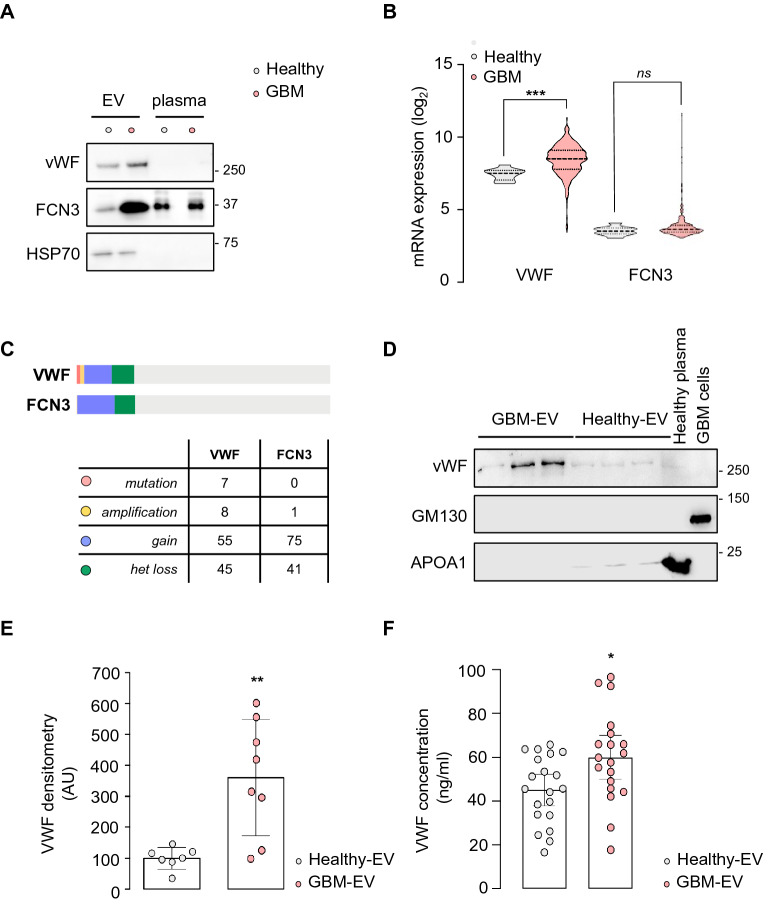
VWF is transported in circulating EVs from GBM patients. (**A**) Equal volume of protein lysates from EV fractions, obtained with SEC followed by ultracentrifugation, and their corresponding plasma, from one GBM patient and one healthy donor, were separated by SDS-PAGE and analyzed by immunoblot for VWF, FCN3, and HSP70. Blots are representative of n = 3. (**B**) mRNA expression of von Willebrand factor (VWF) and ficolin-3 (FCN3) in healthy subjects and GBM patients estimated from The Cancer Genome Atlas (TCGA, n > 500 for GBM patients). (**C**) Anomalies currently reported in VWF and FCN3 genes. (**D**) Equal volume of protein lysates from EV fractions (3 GBM and 3 Healthy) were separated by SDS-PAGE and analyzed by immunoblot for VWF, GM130 (putative intracellular membrane protein contaminant), and Apolipoprotein A1 (APOA1, plasmatic protein). Healthy donor plasma and total cell lysate from GBM cells serve as controls. Blots are representative of n > 7 individual samples. (**E**) Densitometric analysis was performed on 7 and 8 independent EV preparations from Healthy and GBM liquid biopsies, respectively. (**F**) Concentration of VWF in plasmatic EVs from healthy donors and GBM patients (n = 20). Mann–Whitney test, *p < 0.05, **p < 0.01, ***p < 0.001. S.D. are shown in panel E and S.E.M. in panel F.

We then explored more in-depth the potential of these two hits. First, both VWF and FCN3 were detected at high level by western-blots in the EV fractions isolated from the plasma of GBM patients, as compared to EVs from healthy donors (Fig. 4A). In addition, while plasma contained significant amount of FCN3, VWF was barely visualized, suggesting a selective enrichment of VWF in circulating EVs (Fig. 4A). In keeping with this idea, data mining of The Cancer Genome Atlas (TCGA) further unveiled that the RNA expression of *VWF*, but not of *FCN3*, was heightened in GBM patients (Fig. 4B). Moreover, several mutations were documented in the *VWF* gene in GBM patients, as well amplification, pointing again to the clinical interest of monitoring VWF expression (Fig. 4C). In this context, western-blot analysis showed higher VWF levels in EVs isolated from GBM plasma, as compared to healthy donors (Fig. 4D, E). This was further confirmed with ELISA (Fig. 4F). Indeed, VWF protein was significantly higher in EVs from GBM patient blood (mean concentration 59.6 ng/ml, n = 20) than from healthy donors (mean concentration 45.7 ng/ml, n = 20), at the time of diagnosis (Fig. 4F). Our data thus support the notion that EVs from GBM patients are enriched with selective protein cargos that can be further surveyed in circulating EVs, together with vesiclemia.

## Discussion

Seminal studies highlighted extracellular vesicles (EVs) as linchpin tools for tumor growth, survival and therapeutic refractoriness, as they carry and presumably disperse oncogenic material within the GBM ecosystem, as well as throughout the organism. Additionally, recent reports unveiled the presence of EVs into accessible body fluids including cerebrospinal fluid, plasma, and urine. In this context, circulating EVs have been thought to reflect valuable information about the evolving tumor while potentially act as a wide platform for non-invasive biomarkers that might assist in both GBM diagnosis and management. Notwithstanding, the evolution of plasmatic EV profile alongside tumor progression remains uncertain, making standardized information gathered at critical points of GBM progression required.

We assessed here circulating EVs as potential signatures of GBM. Often diagnosed at an advanced stage, there is not, nowadays, any readily markers able to screen for this pathology, to follow its evolution and relapse, and to monitor therapeutic efficacy and individual response to treatment. Recent works have highlighted potential translational applications of EVs in GBM. For instance, the specific RNA miR-21 was found in higher concentration in purified EVs from plasma and cerebrospinal fluid of patients with GBM^5,16^. Earlier studies proposed to block EV secretion and therefore halt tumor growth^17–20^. The potential involvement of EVs in other cancers is also largely explored and provides a promising area of research. Notably, EVs has been suggested as hallmarks in breast, pancreatic, and prostate cancers^10^. In this study, we brought a further comprehensive view on the quantitative and qualitative aspects of circulating EVs at different time course of the tumor management (*i.e.* upon diagnosis, during treatment, and at relapse). Vesiclemia was reported in particles/ml and characterized by at least two different and complementary methods, including single particle analysis techniques (herein interferometry light microscopy) and biochemical techniques, such as ELISA. Electron microscopy, with close-up and wide field provided, was performed to complete EV characterization. According to a standardized and reproducible protocol for EV separation from peripheral blood, we firstly reiterated that the vesiclemia was higher in newly diagnosed GBM patients in comparison with healthy subjects^3,4^. Moreover, plasmatic EVs were found to be smaller in tumor patients, while sharing similar circular morphology, suggesting a potential shift in EV biogenesis and/or cellular source. We further highlighted a quantitative impact of the initial radio-chemotherapy on circulating EVs associated with a stabilizing effect of the 6-month chemotherapy on the vesiclemia. Strikingly, our results stressed out a potential correlation between plasmatic EV abundance and GBM relapse, as 6 out of 7 patients displayed a rise of the vesiclemia upon tumor recurrence. Thus, our data support the idea that circulating EV profile quantitatively fluctuates throughout tumor natural evolution, corroborating recent works from Osti et al*.* where vesiclemia was found to be reduced after resective surgery, while increased upon tumor relapse^4^. Combined, our data strengthen the discovery that circulating EV load might reflect tumor burden. However, larger cohorts are essential to underpin these promising findings. Furthermore, standardized protocols of sampling, storage and analysis must be developed to ensure the reproducibility of the experiments and achieve robustness compatible with clinical routine.

In this work, proteomic analysis unmasked enriched cargos in circulating EVs from GBM patients. Indeed, while principal component analysis demonstrated a clear segregation between tumor and healthy samples. Although a majority of proteins was shared in both conditions, two of them, namely von Willebrand factor (VWF) and ficolin-3 (FCN3), were found to be higher in EVs from GBM patients. In line with these findings, VWF was significantly enriched in plasmatic EVs, as compared to the corresponding plasma of GBM patients, unlike FCN3 which was highly detected in both EVs and plasma (Fig. 4A). Besides, only VWF mRNA expression was found elevated in GBM patients when interrogating TCGA databases, in contrast to FCN3 expression. This is in agreement with earlier findings that also highlight the theranostic value of VWF in brain tumor patients^21,22^. This could reflect EV-mediated tumor pro-angiogenic signaling contributing to GBM major neovascularization activity^6,7^, but also potentially underlie the clinical pro-thrombotic status of cancer patients. To further assess circulating EVs as a non-invasive biomarker of GBM progression, proteomic analysis must be completed with transcriptomic, metabolomic and lipidomic studies to gain more insights on plasmatic EV qualitative aspect and its variation alongside tumor management. Moreover, the identification of a specific molecular signature discriminating between either plasmatic EVs emanating from GBM cells or EVs resulting from pathophysiological release would represent a major step forward. However, this purpose remains complex and highly challenging, as for now no validated marker can track tumor EVs when unleashed in the bloodstream or segregate GBM according to histological/molecular subtypes (*i.e.* proneural, mesenchymal, and classical). Finally, functional studies need to address the role of circulating EVs on different cell types composing GBM microenvironment and brain parenchyma, such as endothelial cells, neurons and astrocytes. In keeping with this idea, future investigations may help in deciphering whether treatments mobilize different types and/or sources for the circulating EV reservoir. One of the biggest challenges that remains to be addressed is therefore the ability to trace the cellular origin of this heterogeneous pool of plasmatic EVs. This should ultimately help in establishing EVs as key messengers hijacked by both tumor and stromal cells to the tumor own’s benefit.

In conclusion, our findings bring new insights on the characterization of circulating EVs from GBM patients and highlight plasmatic EVs as highly promising candidates for GBM bench-to-bedside research.

## Methods

### Ethic statement and plasma samples

Informed consents were obtained from all patients prior to sample collection in the frame of their medical exams. This study was reviewed and approved by the institutional review boards of Angers Hospital (CHU Angers, Angers, France) and Integrated Center for Oncology (ICO, Saint Herblain, France) and performed in accordance with the Helsinki protocol. Two biocollections were established and registered with plasmatic samples at different time course of GBM follow-up. For the French Glioblastoma Biobank (#1476342v2, CHU Angers, Angers, France), 20 liquid biopsies from GBM patients were sampled at the time of diagnosis^23^. For the Integrated Center for Oncology cohort (DC-2015-2457, ICO, Saint Herblain, France), 10 patients with GBM were sequentially sampled at critical points of the Stupp et al. protocol (*i.e.* after resective surgery and upon radio-chemotherapy), during the follow-up and at relapse^24^. In addition, plasma samples from healthy donors were provided by the Etablissement Français du Sang (PLER-NTS2018-021, EFS, Nantes, France). Of note, EVs from healthy donors distributed into three classes of age, (1) < 49 yrs, (2) 50–59 yrs, and (3) > 60 yrs, with mean of particle concentration of 10.1 × 10^9^ ± 3.9, 12.7 × 10^9^ ± 2.8, and 10.1 × 10^9^ ± 3.2, respectively. For all samples, blood was collected on EDTA tubes and centrifuged at 1000*g* for 15 min prior plasma storage at − 80 °C.

### The Cancer Genome Atlas (TCGA) analysis

The Cancer Genome Atlas (TCGA) database was explored via the Gliovis platform (http://gliovis.bioinfo.cnio.es/) ^25^. We interrogated RNAseq data from glioma patients for *VWF and FCN3* expression. Mutations, amplification, gain and heterozygosity loss are also reported.

### Extracellular vesicle separation

Extracellular vesicles (EVs) were separated from plasma by size exclusion chromatography (SEC) using 70 nm Original qEV columns associated with an automatic fraction collector (Izon Science, Lyon, France), according to the manufacturer’s protocol and recommendations from the International Society for Extracellular Vesicles (ISEV)^15^. Briefly, plasma was centrifuged at 10,000*g* for 10 min before loading SEC columns, that allow to eluate particles from different sizes. Of note, qEV7 and qEV8 fractions of 500 µl each, corresponding to EV-eluted fractions, were collected, pooled and harvested for further analysis, while contaminant fractions (*i.e*. from qEV9 to qEV12) were discarded. Alternatively, fractions were concentrated by ultracentrifugation (cryo-EM and proteomics). Samples were ultracentrifugated at 100,000*g* for 2 h using OPTIMA MAX XP ULTRACENTRIFUGE with MLA-130 fixed-angle rotor (Beckman-Coulter, Villepinte, France) and pellets were resuspended in 0.22 µm-filtered PBS. All relevant experimental parameters were submitted to the open-source EV-TRACK knowledgebase (EV-TRACK.org) ^14^. EV-TRACK ID is EV210089.

### Quantitative analysis of EVs

According to recommendations from the ISEV^15^, number of EVs was estimated through two different and complementary procedures. The number of EVs was measured using single particle tracking (Interferometry Light Microscopy, Videodrop, Myriade). Second, the abundance in EVs was also quantified by a biochemical technique using Exo-ELISA CD63 kit (EXEL-ULTRA-CD63-1, SBI, Ozyme, Saint-Cyr-l’Ecole, France) according to the manufacturer’s protocol. Technical triplicates (ILM) and duplicates (ELISA) were performed.

### Cryo-transmission electron microscopy analysis

EVs were separated and concentrated from 1.5 ml of plasma as described above, and then analyzed by cryo-transmission electron microscopy (Microscopy Rennes Imaging Center, Universite de Rennes 1, Rennes, France) using 200 kV Tecnai G2 T20 Sphera microscope (Field Electron and Ion Company), equipped with USC4000 camera and a single axis cryo-holder model 626 (Gatan Microscopy, Pleasanton, CA, USA). Both EV size and circularity index were estimated using ImageJ software v2.0.0-rc-38/1.50b (https://imagej.nih.gov/).

### Flow cytometry analysis

EVs were separated and concentrated from 1 ml of plasma as described above and then analyzed by flow cytometry using CD9 exosome capture beads (ab239685, Abcam, Cambridge, UK) according to the manufacturer’s protocol. PE anti-human CD63 staining (10896786, BioLegend, Paris, France) was next performed. CD9-conjugated bead-coupled EVs were harvested by centrifugation and resuspended in 0.22 µm-filtered PBS. Analysis was performed in triplicate using BD FACS-CANTOII (CYTOCELL platform, SFR François Bonamy, Nantes, France) with 10,000 events recorded for each preparation. Histograms were mounted with FlowJo software version VX (BD, Ashland, OR, USA).

### Immunoblotting

EVs were separated and concentrated from 500 µl of plasma as described above and the pellet was directly lysed in boiling Laemmli for 10 min. Proteins were resolved by SDS-PAGE, transferred onto nitrocellulose membranes and blotted with the following antibodies, all diluted at 1/1000: CD9 (EXOAB-CD9A-1, SBI), CD63 (EXOAB-CD63A-1, SBI), VWF (sc-53466, Santa Cruz), FCN3 (CL7767AP, Cedarlane)**,** Apolipoprotein A1 (sc-376818, Santa Cruz), HSP70 (EXOAB-HSP70A, SBI), and GM130 (ab52649, Abcam) diluted at 1/1000. Membranes were incubated with HRP-conjugated secondary antibodies (Southern Biotech), diluted at 1/5000 and then revealed by chemiluminescence. Acquisitions were performed with Fusion software, version FX7 16.15 (Vilbert Lourmat, Collegien, France). Uncropped blots are available in Fig. S1.

### Mass spectrometry and proteomic analysis

Label free mass spectrometry analysis was performed (Proteom’IC, Institut Cochin, 3P5, Universite Paris Sorbonne, Paris, France). EVs were separated and further concentrated from 1 ml of randomly chosen plasma of 3 GBM patients and healthy donors, as described above. Proteins were lysed and denatured in SDS 2%, 50 mM Tris pH = 8 (5 min at 95 °C), hydrolyzed with trypsin and released peptides were analyzed in triplicate by liquid chromatography with tandem mass spectrometry (LC–MS/MS) using Orbitrap Fusion mass spectrometer (ThermoFisher Scientific, Courtaboeuf, France). Obtained spectra were processed using MaxQuant (https://www.maxquant.org/) and Perseus servers (https://www.maxquant.org/perseus_plugins/) and compared to Swiss-Prot/TrEMBL (https://www.uniprot.org/blast/) banks for protein identification. Label-free quantification was performed on 3 samples in the control and GBM groups, while FDR and number of identified peptides are reported (Supplementary Table S1). Number of valid values (VV) per group (0, 1, 2 or 3) allows the selection of proteins that are considered as expressed (VV of 2 or 3) or absent (VV of 0 or 1). Principal component analysis (PCA) was next performed on 100% VV proteins, while peptides with a *p*-value inferior to 0.01 (t-test) were considered differentially expressed.

### Quantitation of von Willebrand factor (VWF)

VWF concentration was estimated on EV preparation using human VWF ELISA kit (EHVWFX, ThermoFisher Scientific) according to the manufacturer’s protocol. Technical replicates by serial dilution were performed.

### Quantitation of proteins

Protein concentration of each qEV fraction was estimated by BCA protein assay according to the manufacturer’s protocol (ThermoFisher Scientific). Technical replicates by serial dilution were performed.

### Statistical analysis

Statistical analyses were performed with Prism v.8.3.0 software using unpaired two-tailed Mann–Whitney test (non-parametric test) and ordinary one-way analysis of variance (ANOVA). For all the experiments, a *p*-value inferior to 0.05 in considered significant.

## Supplementary Information

Supplementary Table S1.

Supplementary Information 1.
