# Supplementary material for: The von Willebrand factor stamps plasmatic extracellular vesicles from glioblastoma patients
Source: Sci Rep. 2021 Nov 23;11:22792. doi: 10.1038/s41598-021-02254-7 (PMC8611030; doi:10.1038/s41598-021-02254-7)
Supplement: Supplementary file 2 — Supplementary Information 1. [file 41598_2021_2254_MOESM2_ESM.pdf]

The von Willebrand Factor stamps Plasmatic Extracellular Vesicles from Glioblastoma Patients

Quentin Sabbagh, Gwennan André-Grégoire, Carolina Alves-Nicolau, Aurélien Dupont, Nicolas Bidère, Emmanuel Jouglar, Laëtitia Guével, Jean-Sébastien Frénel, Julie Gavard

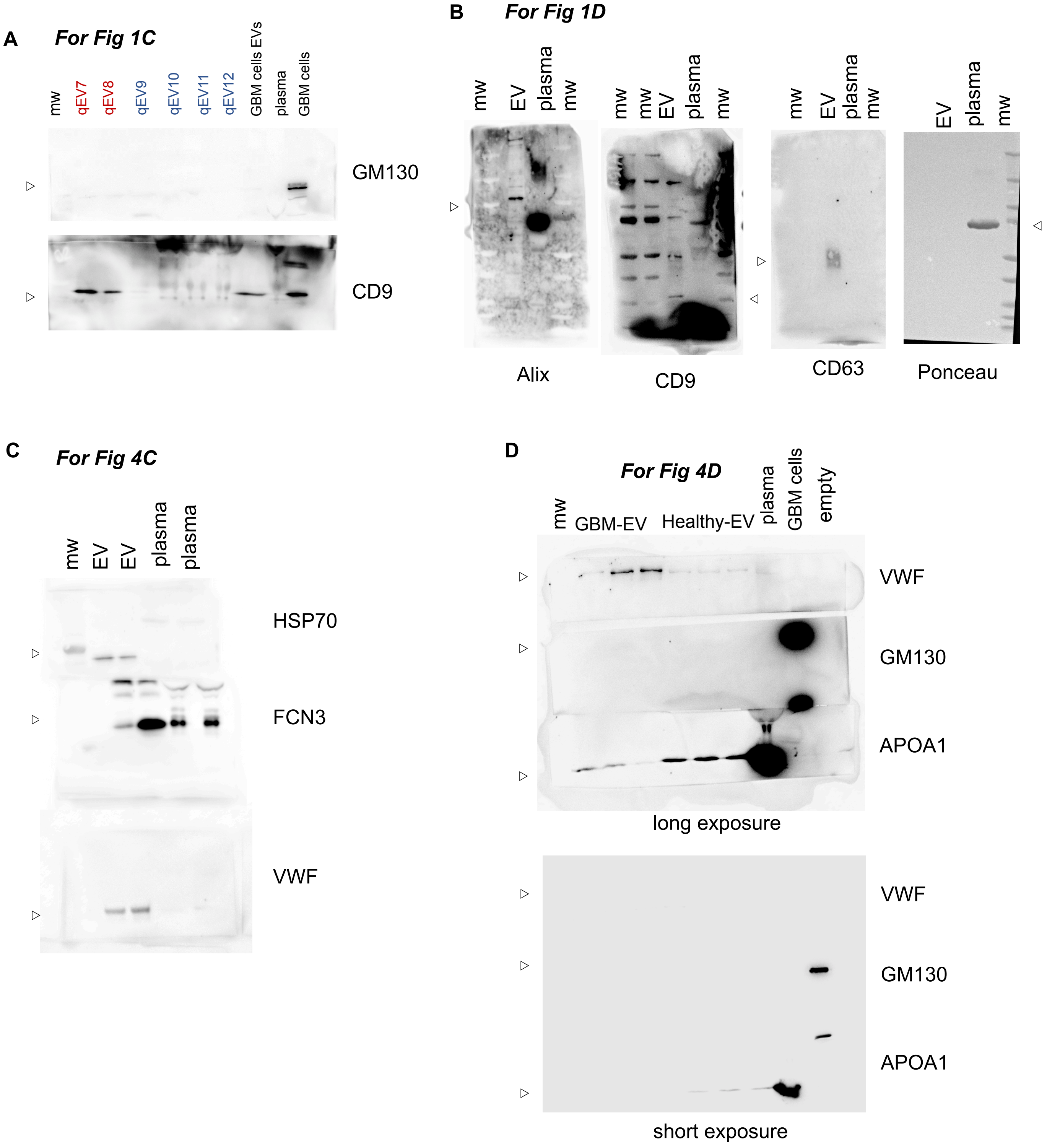

Figure S1. Uncropped scans for Figures 1C, 1D, 4C, and 4D.
Uncropped blots are provided, either as membranes cut before antibodies hybridization (panels A, C, D), and full scans of membranes (panel B).
mw: molecular weight ladder, EV: extracellular vesicles, qEV: size exclusion chromatography fractions, GBM: glioblastoma.

name	peptide count	score	coverage (%)
**ANAX7**	4;3;5;0;0;0	6.9	12.2
**PDLIM1**	0;1;1;8;12;9	83.3	55.5
**ANK1**	0;0;0;4;20;18	67.3	13.2
**EPB42**	0;0;1;2;9;8	28.7	17.0
**CALD1**	0;0;2;10;16;11	49.8	33.1
**CTTN**	1;0;0;3;12;11	74.4	35.5
**TMEM40**	0;0;0;2;5;4	18.3	26.2
**HSD17B10**	0;0;1;2;2;2	4.2	9.6

Supplementary Table S2. Exclusively Expressed Proteins from the label-free proteomic analysis in 3 control (1;2;3) and GBM (1;2;3) plasmatic EV fractions.

name	peptide count	score	coverage (%)
**TAGLN2**	4;6;6;3;6;4	97.4	44.5
**CSTA**	5;4;0;4;2;3	16.5	67.3
**YWHAG**	5;7;8;6;8;8	67.5	35.2
**YWHAE**	11;13;13;7;10;9	36.4	55.7
**YWHAB**	8;11;10;8;8;9	47.0	48.8
**PPIA**	7;9;7;6;6;3	38.9	53.3
**FCN3**	3;1;3;13;13;12	85.4	53.8
**VWF**	26;27;57;99;101;93	323	44.0

Supplementary Table S3. Differentially Expressed Proteins from the label-free proteomic analysis in 3 control (1;2;3) and GBM (4;5;6) plasmatic EV fractions.
